# Leakage Detection Using Distributed Acoustic Sensing in Gas Pipelines

**DOI:** 10.3390/s25164937

**Published:** 2025-08-10

**Authors:** Mouna-Keltoum Benabid, Peyton Baumgartner, Ge Jin, Yilin Fan

**Affiliations:** 1 Petroleum Engineering Department, Colorado School of Mines, Golden, CO 80401, USA; mbenabid@mines.edu (M.-K.B.);; 2 Geophysics Department, Colorado School of Mines, Golden, CO 80401, USA; gjin@mines.edu

**Keywords:** distributed acoustic sensing, distributed fiber optic sensing, gas pipeline monitoring, leakage detection, internal fiber deployment, buried pipelines, flow-induced vibrations, vibration analysis, time-domain signal processing, spectral analysis

## Abstract

This study investigates the performance of Distributed Acoustic Sensing (DAS) for detecting gas pipeline leaks under controlled experimental conditions, using multiple fiber cable types deployed both internally and externally. A 21 m steel pipeline with a 1 m test section was configured to simulate leakage scenarios with varying leak sizes (¼”, ½”, ¾”, and 1”), orientations (top, side, bottom), and flow velocities (2–18 m/s). Experiments were conducted under two installation conditions: a supported pipeline mounted on tripods, and a buried pipeline laid on the ground and covered with sand. Four fiber deployment methods were tested: three internal cables of varying geometries and one externally mounted straight cable. DAS data were analyzed using both time-domain vibration intensity and frequency-domain spectral methods. The results demonstrate that leak detectability is influenced by multiple interacting factors, including flow rate, leak size and orientation, pipeline installation method, and fiber cable type and deployment approach. Internally deployed black and flat cables exhibited higher sensitivity to leak-induced vibrations, particularly at higher flow velocities, larger leak sizes, and for bottom-positioned leaks. The thick internal cable showed limited response due to its wireline-like construction. In contrast, the external straight cable responded selectively, with performance dependent on mechanical coupling. Overall, leakage detectability was reduced in the buried configuration due to damping effects. The novelty of this work lies in the successful detection of gas leaks using internally deployed fiber optic cables, which has not been demonstrated in previous studies. This deployment approach is practical for field applications, particularly for pipelines that cannot be inspected using conventional methods, such as unpiggable pipelines.

## 1. Introduction

Ensuring the structural integrity of oil and gas pipelines remains a critical priority for the energy industry. Pipelines form the backbone of the global energy infrastructure, enabling the safe and efficient transport of hydrocarbons across long distances [1]. In the United States alone, there are approximately 3.3 million miles of regulated pipelines, which collectively transport about 64% of the nation’s energy commodities. Of these, approximately 2.78 million miles are regulated gas pipelines, many of which traverse remote or environmentally sensitive areas [2]. Failures in these systems, particularly in the form of leaks, can result in environmental degradation, operational disruptions, and significant threats to public safety [3,4,5]. As pipeline infrastructure continues to age and energy security becomes increasingly important, the industry requires advanced monitoring technologies that support early warning and real-time surveillance [6]. Rapid identification of abnormal pipeline behavior is essential for minimizing the impact of potential failures, improving emergency response, and ensuring compliance with environmental and safety regulations.

Pipeline integrity assessment has traditionally relied on methods such as in-line inspection (ILI) tools, hydrostatic pressure testing, and pressure-monitoring systems, each offering distinct capabilities and limitations [7]. ILI tools, commonly referred to as “smart pigs,” are deployed periodically to identify internal anomalies such as corrosion, wall thinning, or geometric deformation. While effective for evaluating structural conditions, these tools provide only intermittent snapshots of pipeline health, require significant operational planning, and are unsuitable for continuous or remote monitoring [8,9]. Hydrostatic testing involves pressurizing the pipeline beyond its operating limits to verify its mechanical integrity and expose weaknesses that may eventually result in leakage. However, this technique necessitates pipeline downtime, uses large volumes of water, and produces test water that often contains trace hydrocarbons, requiring treatment before disposal. It may also accelerate the growth of pre-existing flaws [10]. Pressure drop monitoring and mass balance calculations are commonly employed for leak detection, but they are often less sensitive to small leaks and provide only coarse estimates of leak location [11]. Visual inspections and satellite monitoring may detect surface evidence of leakage or external threats but are labor-intensive and generally limited to identifying large or noticeable leaks [12]. Although these conventional approaches contribute to maintaining pipeline integrity, they are often reactive, resource-intensive, and incapable of providing the continuous, high-resolution surveillance needed to detect and localize leaks early. These limitations have prompted growing interest in alternative monitoring technologies capable of continuous and distributed detection, particularly in inaccessible or high-risk environments.

**Figure 1:**
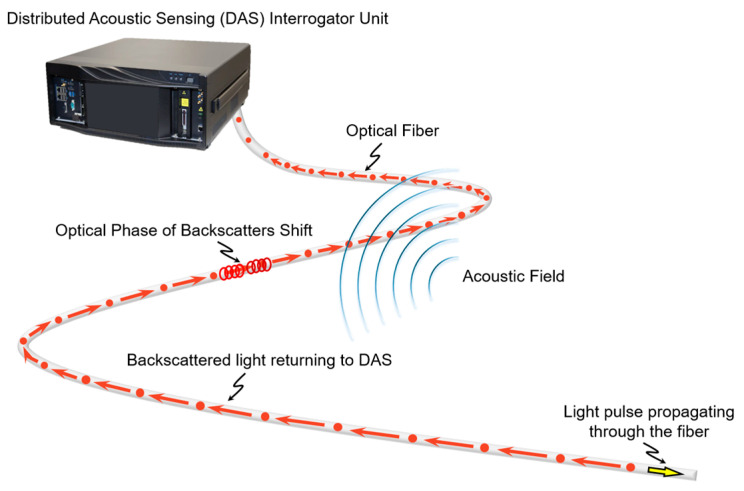
Conceptual diagram of a DAS system. Coherent laser pulses travel through the optical fiber, and external vibrations induce phase shifts in the Rayleigh backscattered light. These shifts are measured to provide spatially resolved vibration-induced strain-rate data for detecting pipeline leaks.

Distributed fiber-optic sensing (DFOS) has become an increasingly valuable technology for pipeline integrity monitoring [13], particularly in situations where conventional methods are impractical or unavailable. These systems utilize standard telecommunication fibers connected to an interrogator unit to provide high-resolution, distributed measurements along the length of the pipeline. DFOS technologies can cover long distances, often monitoring more than 50 km of fiber [14], which makes them especially suitable for remote or difficult-to-access segments of pipeline infrastructure. Unlike traditional inspection methods, DFOS enables continuous and real-time monitoring without requiring operational shutdowns. This capability supports early detection of anomalies and allows timely intervention before failures occur. Distributed Acoustic Sensing (DAS), a widely applied DFOS technique, has shown strong capability in detecting pipeline leaks by capturing acoustic and vibrational energy along the fiber [15,16,17]. In DAS systems, coherent laser pulses are transmitted through the fiber, and phase shifts in the Rayleigh backscattered light are measured and analyzed to obtain spatially resolved strain rate data [18]. This method enables continuous identification of dynamic disturbances in the pipeline, including those generated by fluid leakage, with high spatial resolution (Figure 1). DAS is an effective solution for pipelines where conventional monitoring approaches cannot be applied, such as unpiggable or physically inaccessible networks.

Recent studies have demonstrated the capability of DAS for detecting pipeline intrusions and leakage-related vibrations. Tejedor et al. (2017) [19] demonstrated the use of DAS signals to detect pipeline intrusion events, while Stajanca et al. (2018) [20] showed that DAS signals are sensitive to vibrations caused by leakage. However, these studies encountered difficulties in distinguishing vibrations originating from the pipeline infrastructure from those associated with leaks, particularly under high-pressure and high-flow conditions. The deployment method of the sensing fiber also plays a critical role in the cost and effectiveness of DAS-based monitoring. Helically wrapping the fiber around the pipeline enhances spatial resolution and sensitivity but increases installation costs and limits sensing range [21]. Attaching the fiber in a straight configuration on the pipe’s exterior offers a more practical approach but may be unfeasible for existing buried pipelines. Internally deploying sensing cables by pumping or dragging them through the pipeline presents a cost-effective option; however, it introduces challenges in separating flow-induced noise from actual leakage signals [22].

This study evaluates the performance of DAS for detecting gas pipeline leaks under controlled laboratory conditions by examining the effects of pipeline installation method, leak size and orientation, flow rate, fiber cable type, and deployment approach. Experiments were conducted using a 21 m steel pipeline with a dedicated 1 m test section in the middle, designed to accommodate leaks of varying sizes (¼”, ½”, ¾”, and 1”) and orientations (top, side, bottom). Gas flow was tested at five velocities (2, 6, 10, 14, and 18 m/s). The pipeline was configured under two installation scenarios: a supported setup using tripods and a buried configuration covered with sand to simulate different field-relevant conditions. Four fiber deployment methods were evaluated, including one externally mounted straight cable and three internally deployed cables with distinct structural designs. DAS data were analyzed using both time-domain vibration intensity and frequency-domain spectral methods to assess leak detectability. The results highlight the combined influence of pipeline configuration, leak characteristics, and fiber deployment on detection sensitivity. This work provides new experimental evidence supporting the feasibility of using internally deployed fiber-optic cables for gas leak detection, offering a practical and scalable solution for monitoring unpiggable or inaccessible pipelines where traditional inspection methods are not viable.

The remainder of this paper is organized as follows: Section 2 describes the experimental facility and DAS data acquisition and processing methods. Section 3 presents the results for both supported and buried pipeline configurations, including standard deviation visualization, leak detectability analysis by size, location, and flow rate, and spectrum analysis. Section 4 provides the discussion, and Section 5 concludes the study with key findings.

## 2. Materials and Methods

### 2.1. Experimental Facility and Setup

**Figure 2:**
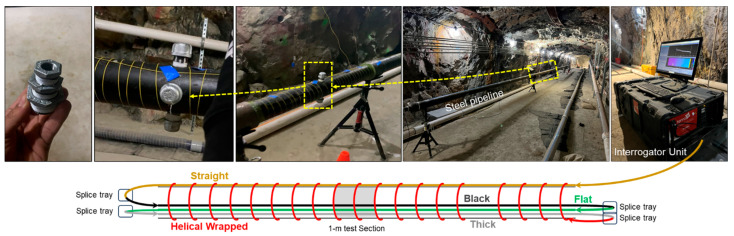
Photographs of the experimental setup and a schematic illustrating the fiber optic cable deployment along the steel pipe. The cable placement sequence is external straight, internal black, internal flat, internal thick, and external helical-wrapped.

**Figure 3:**
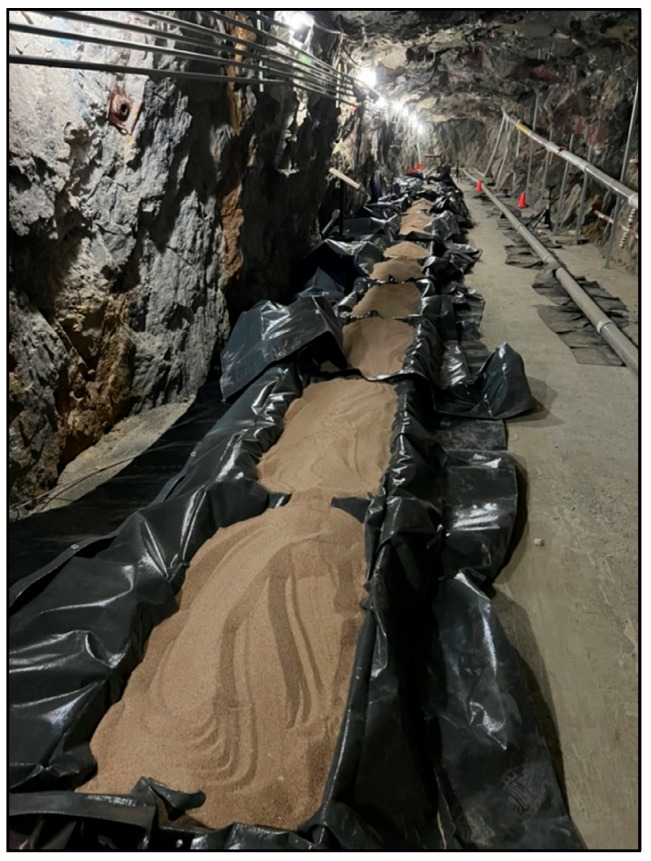
Photographs of the experimental setup and a schematic illustrating the fiber optic cable deployment along the steel pipe.

**Figure 4:**
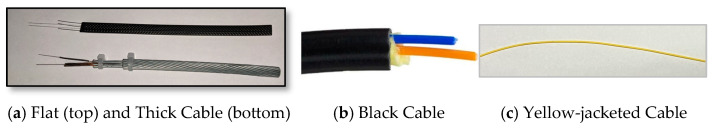
Photographs of the tested fiber optic cables.

To evaluate leakage detection using DAS in a controlled environment, a steel pipeline was constructed at Edgar Mine, an experimental mine owned by Colorado School of Mines. As shown in Figure 2 and Figure 3, the pipeline is located inside a tunnel, offering a low-noise environment that minimizes ambient interference and measurement uncertainty. The facility maintains stable conditions throughout the year, with a dry atmosphere and a constant temperature of 54 °F. The experimental setup consists of a 21 m-long API 5L Grade X65 steel pipeline with a 4-inch internal diameter (Federal Steel Supply, Inc., Boston, United States). A 1 m central section was designed for leakage simulation, with 1-inch holes drilled at the top, side, and bottom to represent different leak orientations. Leak sizes were varied using galvanized malleable iron fitting reducers threaded into these holes, producing diameters of ¼”, ½”, ¾”, and 1”. Leakage experiments were conducted under two pipeline configurations: (i) a supported setup, where the pipeline was mounted on five tripods evenly distributed along its length, as shown in Figure 2; and (ii) a buried setup, where the pipeline was placed inside a series of wooden containment boxes measuring 2.44 m in length, 0.61 m in width, and 0.28 m in height, each subdivided into two equal compartments of approximately 1.22 m length. These compartments defined individual burial segments along the pipeline. A washed and graded silica sand with minimal fines was used as the burial medium, with a typical loose bulk density of approximately 1600 kg/m^3^. The sand was used in its as-received dry condition without added moisture. Approximately five 23 kg bags of sand were added to each compartment, providing bedding beneath the pipe and cover above it. The sand was poured and roughly leveled to ensure uniform coverage around the pipe, but it was not mechanically compacted, as shown in Figure 4.

Compressed air was used in all experiments, with properties of 1.2 kg/m^3^ density and 1.83 × 10^−5^ Pa·s viscosity at approximately 19 °C. Gas velocities of 2, 6, 10, 14, and 18 m/s were tested. The pipe internal pressure was below 20 psi. Control experiments, conducted at the same flow rates without leakage, served as baselines for comparison. The pipeline was instrumented with an airflow meter, pressure sensors (at the inlet and leakage section), and air-controlled valves for precise flow regulation and velocity calculation.

To assess the sensitivity and detection performance of different fiber optic cables, multiple single-mode optical fibers were installed along the pipeline (Figure 4). In the supported configuration, three internal cables were deployed: a black cable, a flat cable, and a thick cable resembling a wireline. The black and flat cables are standard outdoor distribution fibers. Additionally, a telecommunications-grade yellow-jacketed fiber was externally affixed in a straight configuration. A helically wrapped, yellow-jacketed fiber with 3 cm spacing was initially installed (see Figure 2) but was later excluded from the analysis due to multiple fiber breakages during the experiment that prevented data acquisition. This incident highlights the fragility of thin yellow-jacketed fibers and their potential limitations in field applications. All cables used are single-mode, standard telecommunications fibers. The black cable has a diameter of approximately 5.5 mm, the thick cable 6.0 mm, the flat cable 4.3 mm × 1.7 mm, and the yellow-jacketed fiber 0.9 mm. The internal fibers were inserted through a rubber-sealed fitting at the pipeline inlet, maintaining pressure integrity while allowing flexible movement. This setup facilitated the convenient replacement of the 1 m test section. Internal cables lay freely inside the pipe without physical attachment to the inner wall. From the interrogator, the fiber connection sequence was externally taped straight cable, then black, flat, and thick cables. Fiber splicing was performed at both ends using splicing trays, forming a continuous optical path length of approximately 220 m. In the buried configuration, the fiber deployment remained the same, except that the thick cable was omitted due to cable availability constraints following multiple splicing attempts. As a result, data from the buried configuration excludes the thick cable.

### 2.2. DAS Data Acquisition and Processing

In this study, DAS data were collected using a Terra15 interrogator unit (Terra15 Technologies Pty Ltd, Perth, Australia), which captures high-frequency vibrations along the sensing fiber and allows gauge length to be assigned during post-acquisition processing [23]. Data were acquired with a temporal sampling frequency of 14.39 kHz and a spatial sampling interval of 0.82 m, while the interrogator operated at a pulse rate of 22.63 kHz, and a pulse length of 1.63 m. Preprocessing included converting velocity measurements to strain rate. To enhance spatial resolution, the smallest available gauge length, equal to the 0.82 m spatial step, was applied.

**Figure 5:**
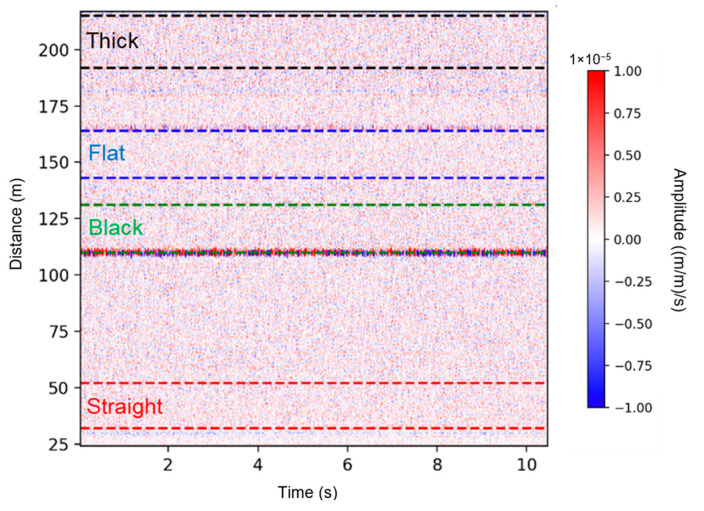
A 10 s segment of raw DAS data recorded during the bottom leakage experiment in the supported pipeline, conducted at a flow velocity of 10 m/s with a 1-inch leak. The dashed lines indicate fiber segmentation, marking the start and end positions of each cable: red for the straight external cable, green for the black cable, blue for the flat cable, and black for the thick cable.

Figure 5 presents 10 s of raw DAS data recorded during a bottom leakage experiment in the supported pipeline, conducted at a flow velocity of 10 m/s with a 1-inch hole size. The figure also illustrates the segmentation of the sensing cables. In the supported configuration, all four fiber optic cables were interrogated sequentially, with each cable spliced to the preceding one. In the buried configuration, the thick cable was excluded, and the fiber start and end positions varied due to re-splicing adjustments. Table 1 summarizes the fiber segment positions and flow directions for both pipeline configurations.

Preprocessing of the raw DAS data was necessary before conducting the analysis. Several processing techniques were applied to enhance leakage detection and quantify anomalies in the acquired signals. These included standard deviation computation and spectral analysis using the Fourier transform. Most of the algorithms and code used in this study are available in the open-source DASCore Python library (v0.1) [24].

#### 2.2.1. Standard Deviation Processing and Vibration Intensity Quantification

Due to its high spatial and temporal resolution, raw DAS data are substantially large. For example, a three-hour recording produced approximately 351 GB of data. To facilitate long-duration leakage analysis, it was necessary to reduce the dataset size. This was achieved through standard deviation (SD) processing, where the data were divided into one-second intervals, and the SD was computed for each channel per second. These values were then concatenated into a continuous dataset with a 1 Hz sampling rate, significantly down-sampling the original data by a factor of 14,390, corresponding to the initial acquisition rate. To enhance visualization and analysis, the SD values were converted to decibels (dB) using a reference value of 1. Since the SD over a given time window is proportional to the dynamic energy of the vibrations, this transformation preserved critical information about vibration intensity along the fiber while achieving substantial data reduction. SD processing made it feasible to visualize large datasets, whereas raw data would have been impractical to handle due to its size.

To quantify vibration anomalies at leakage points, the average vibration intensity was computed over a four-minute window during the middle of each experiment. This ensured the analysis captured a representative segment of vibration activity. The initial and final 30 s were excluded to avoid the transient period associated with flow rate adjustments. By averaging the intensity values, a distinct vibration profile was established for each fiber optic cable. These profiles were then converted into decibels (dB) to facilitate comparisons across different cables and experimental conditions.

#### 2.2.2. Spectrum Analysis

To further investigate the impact of leakage on cable vibrations, a Fourier spectrum analysis was conducted on the DAS measurements. The primary objective was to identify frequency ranges associated with leakage-induced signals. In this process, the raw DAS data were divided into 10 s segments, and the amplitude spectrum was calculated for each channel along the sensing cables. To enhance statistical reliability, spectra from four-minute intervals within the same experiment were stacked and averaged. The final spectra were structured as 2D arrays, where columns represent spectral amplitudes at different frequencies and rows correspond to individual channels along the fiber. A detailed discussion of the results is provided in the following section.

## 3. Results

This section presents a detailed analysis of the experimental data for both supported and buried pipeline configurations. It includes standard deviation visualization, leakage detectability across various fiber cables, and frequency-domain spectral analysis. Each subsection highlights key findings and demonstrates how the applied methodologies successfully localize leakage with sensitivity influenced by experimental parameters such as leak characteristics, flow conditions, and pipeline installation configuration.

### 3.1. Standard Deviation Visualization

**Figure 6:**
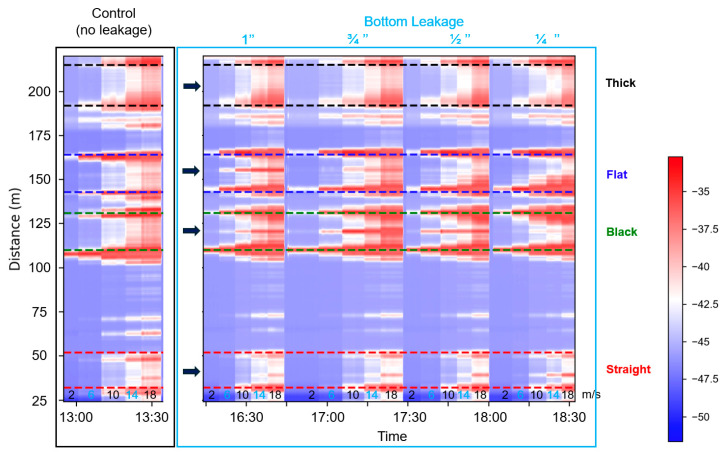
Magnitude of standard deviation values in straight external (red), black (green), flat (blue), and thick (black) fiber sections during the control, and bottom leakage experiments with different hole sizes in the supported pipeline setup. The flow velocities inside the pipe change from 2, 6, 10, 14, and 18 m/s (The gas velocities are indicated at the bottom of the figure). The arrows indicate the leakage location (at the center of all cable sections).

Figure 6 presents the SD data attributes for the supported pipeline during an experiment involving bottom leakage holes. The results reveal that higher SD values (depicted in red) correspond to increased vibration intensity at specific times and locations along the sensing fiber, particularly near the leakage site at the pipe’s center [25]. The experiment began with a control test (no leakage), followed by sequential leakage tests with hole sizes of 1”, ¾”, ½”, and ¼” at flow velocities of 2, 6, 10, 14, and 18 m/s. As expected, higher flow rates induced stronger vibrations across all cable types, with the most pronounced anomalies appearing near the leakage point. Among the sensing cables, the black and flat internal cables exhibited the highest sensitivity, capturing clear vibration signatures even for smaller hole sizes. In contrast, the external straight-taped cable showed a weaker response, and the thick cable was the least sensitive, likely due to its heavy structure reducing responsiveness to flow-induced vibrations.

**Figure 7:**
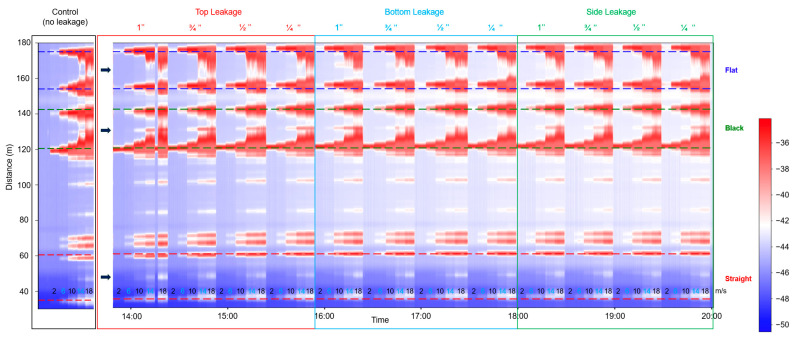
Magnitude of standard deviation values in straight external (red), black (green), and flat (blue) fiber sections during the control, and leakage experiments (top, bottom, and side) with different hole sizes in the buried pipeline setup. The arrows indicate the leakage location (at the center of all cable sections).

Figure 7 presents the SD data attributes for the buried pipeline, in which the same leakage conditions and flow velocities were tested as in the supported setup. In addition to the bottom leakage, the data also includes top and side leakage tests. The general trend observed in the supported configuration, that higher flow rates produce stronger vibration intensities, remains evident. However, compared to the supported case, the detection sensitivity is notably reduced in the buried pipeline, as indicated by the data from the straight and flat cables. Vibration anomalies are less pronounced, and the distinction between leakage and non-leakage conditions is less clear. This reduction in sensitivity is likely due to burial-induced damping effects, which attenuate vibration transmission and lower the amplitude of the DAS signal. These findings underscore the significant influence of pipeline installation on DAS-based leakage detection. Interestingly, the black cable still shows a strong response at the leakage point, particularly in the top and side leakage tests. However, subsequent frequency-domain analysis suggests that this response may be influenced by factors other than leakage itself. This will be discussed further in the spectrum analysis in Section 3.3.

### 3.2. Leakage Detection Sensitivity Across Different Sensing Cables Based on Vibration Intensity

This section illustrates the leakage detectability of different sensing cables based on the vibration intensity, considering the effects of leak size, leak position, flow rate, and pipeline installation configurations.

#### 3.2.1. Based on Leak Size and Position

**Figure 8:**
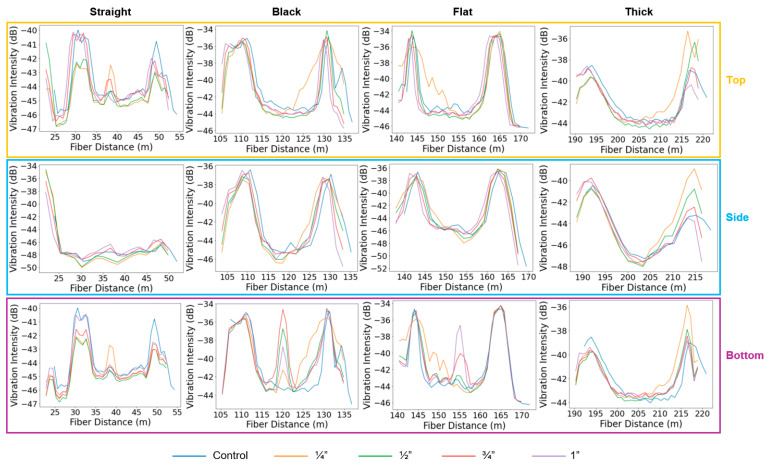
Vibration intensity profiles along the straight external (**left**), black (**center-left**), flat (**center-right**), and thick (**right**) fiber sections during the top, side, and bottom leakage experiments in the supported pipe. The different curves correspond to leakage hole sizes of ¼”, ½”, ¾”, and 1”, at a flow velocity of 10 m/s.

Figure 8 presents the vibration intensity profiles along all fiber cables in the supported pipeline configuration during the control experiment and the leakage experiments conducted at the top, side, and bottom positions, using four leak sizes (¼”, ½”, ¾”, and 1”) at a flow velocity of 10 m/s. It highlights the sensitivity of the various sensing cables to different leak sizes and positions. In general, leakage at the bottom position exhibited the most pronounced vibration anomalies. The straight external cable, as well as the internal black and flat cables, were able to detect a vibration anomaly when the leak size exceeded ½”, showing varying levels of sensitivity depending on the leak size. In contrast, the thick cable, being notably heavier, showed the lowest sensitivity to vibrations induced by the leaks, regardless of size or location.

In experiments where leaks were introduced at the top and side of the steel pipe, identifying vibration anomalies became more challenging. Across all cables and leak sizes, no clear vibration anomaly was observed at the leak position, with one exception: the straight cable detected the top-positioned leak when the leak size was ¾” or larger. This directional sensitivity is attributed to the external placement of the straight cable, which was taped along the top of the pipe and therefore aligned with the leak location. Its coupling and proximity to the leak enhanced its responsiveness, in contrast to the internal cables that were loosely positioned along the internal bottom of the pipe. Notably, the straight cable response to side-positioned leaks differed in the vibration profile shape and pattern compared to the top and bottom experiments. The variation may be attributed to the time gap between experiments, as the top and bottom leaks were recorded on the same day, whereas the side leak data were collected a month later. Over time, the tape securing the external cable may have degraded, leading to altered coupling conditions. In contrast, the internal cables maintained stable coupling conditions across all experiments.

**Figure 9:**
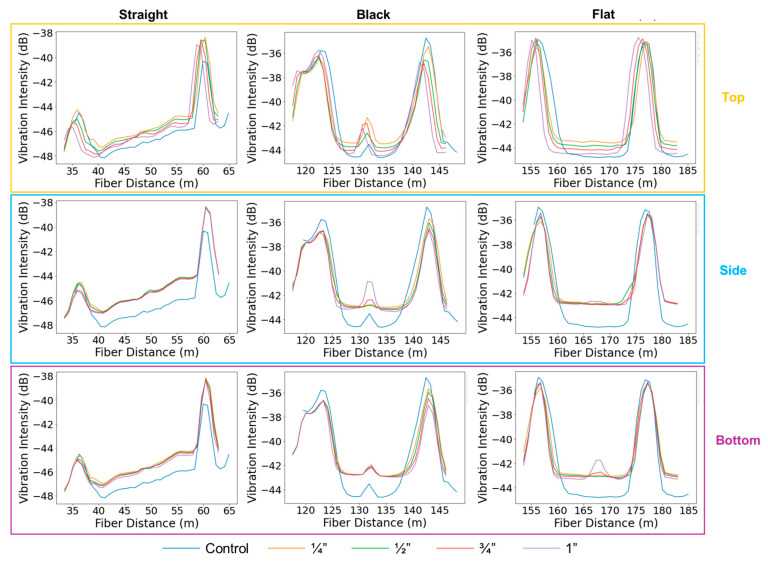
Vibration intensity profiles along the straight external (**left**), black (**middle**), and flat (**right**) fiber sections during the top, side, and bottom leakage experiments in the buried pipe. The different curves correspond to leakage hole sizes of ¼”, ½”, ¾”, and 1”, at a flow velocity of 10 m/s.

In the buried pipeline configuration (Figure 9), overall leakage detectability was significantly reduced compared to the supported setup. The flat cable showed a clear response for bottom-positioned leaks with a 1-inch leak size and exhibited a weaker but noticeable response for the 3/4-inch case, while no response was observed for top or side leaks. The straight cable did not exhibit any measurable response across all tested leak sizes and orientations. These results suggest that leak detectability is largely diminished in the buried configuration, likely due to vibration attenuation caused by the surrounding sand. Consistent with observations from the supported pipe, the flat cable is most sensitive to bottom leaks, likely due to its proximity to the leak location. In contrast, the vibration intensity plots from the black cable do not show a clear trend with leakage position. This phenomenon is further discussed in Section 3.3.

Another interesting observation from Figure 9 is the increasing gap between the control tests (blue curves) and the subsequent leakage tests as the sequence progresses from top and bottom to side leakages. This trend is also reflected in the SD plot shown in Figure 7. It is important to note that the control tests were conducted first, followed by the top, bottom, and side leakage tests. The SD plots indicate that the background vibration intensity gradually increased over time, as reflected by the progressively warmer colors. One possible explanation is splicing degradation, which is supported by the observation that this gap widens from the straight to the flat cable. Other factors, such as minor changes in environmental/operational conditions over time, may also contribute. We acknowledge that further investigation is needed to fully understand this phenomenon.

#### 3.2.2. Based on Flowrate

**Figure 10:**
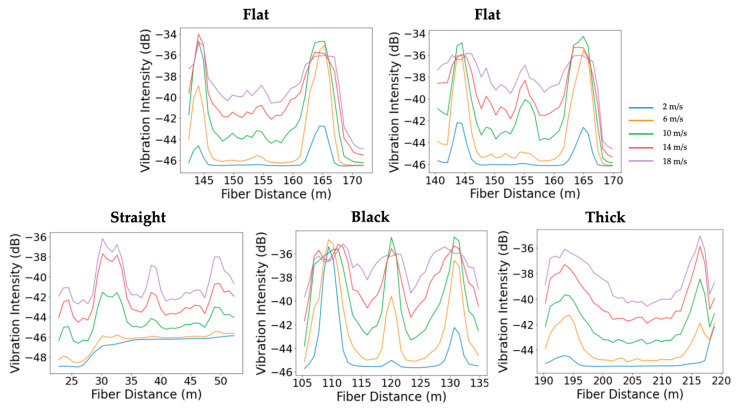
Comparison of leakage detection sensitivity across different fiber cable types during bottom leakage experiments in the supported pipe at various flow velocities. The top left plot shows the flat cable response under no-leakage (control) conditions, while the top right displays its response to a ¾-inch bottom leak. The bottom row presents the responses of the straight, black, and thick cables (left to right), each tested with the same ¾-inch bottom leak.

The sensitivity of leakage detection is also influenced by the gas flow rate within the pipeline. Figure 10 presents the vibration intensity profiles along all the fiber cables for different flow velocities, with a fixed bottom leakage size of ¾”. In the supported configuration, the flat cable under no-leakage conditions (control) shows increasing vibration intensity as flow velocity increases, but no anomaly is observed at the pipe center. When a bottom leak is introduced, a clear vibration anomaly appears at the leak location across all tested flow rates for the straight and flat cables. The thick cable remains unresponsive, consistent with earlier observations. Among the responsive cables, detectability improves with increasing flow velocity, indicating enhanced sensitivity to leakage-induced vibrations at higher flow rates.

**Figure 11:**
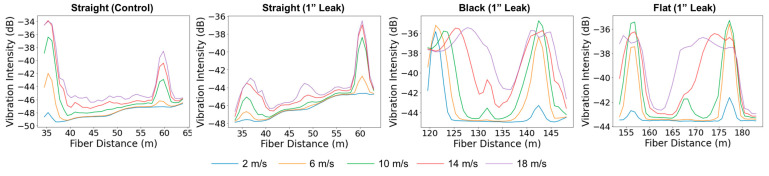
Leakage detection sensitivity of different fiber cable types during bottom leakage experiments in the buried pipeline at various flow velocities. The left plot shows the straight cable response under no-leakage (control) conditions, followed by the responses of the straight, black, and flat cables to a 1-inch bottom leak.

In the buried pipeline configuration (Figure 11), a similar trend is observed. The vibration intensity profiles for all cables investigated are shown at various flow velocities, using a fixed bottom leak size of 1”. In the control case, measured with the straight external cable, the vibration intensity increases with flow velocity, but no anomaly appears at the pipe center. When the 1” leak is introduced, the straight cable, which showed no measurable response at 10 m/s, begins to detect a vibration anomaly at the highest tested flow velocity of 18 m/s. For the black and flat cable, the anomaly starts becoming visible at 10 m/s. These results confirm that increasing gas flow enhances detection capability.

However, the trend is not strictly linear, especially for the internal flat cable. At low flow velocities, the signal from the leakage is weak and may not be detectable. As flow velocity increases, the signal-to-noise ratio improves, allowing the leakage to become visible. Yet, at higher flow velocities, the noise level within the pipe increases more rapidly than the signal, particularly for internal cables directly exposed to the fluid flow. This rise in background noise can reduce the signal-to-noise ratio and make leakage detection more difficult, even when a signal is present. This suggests there is a turning point in flow velocity, beyond which detectability may decline due to increased internal interference. This turning point may vary depending on the specific pipeline operating conditions. It is important to note that our experiments were conducted on a relatively short 21 m steel pipe. The short length amplifies boundary effects, here defined as increased fiber vibrations near the inlet and outlet, where air enters and exits the pipe, resulting in higher background noise at these locations. In contrast, longer field pipelines are expected to exhibit lower relative noise levels and improved signal-to-noise ratios. Therefore, better detectability is anticipated in real-world applications compared to the lab-scale setup.

### 3.3. Leakage Detection Sensitivity Across Different Sensing Cables Based on Frequency Spectrum Analysis

To further characterize the leakage-induced signals, a Fourier transform was applied to the time-domain data, yielding two-dimensional spectral representations. This approach enables the identification of dominant frequency components associated with leakage-induced vibrations.

**Figure 12:**
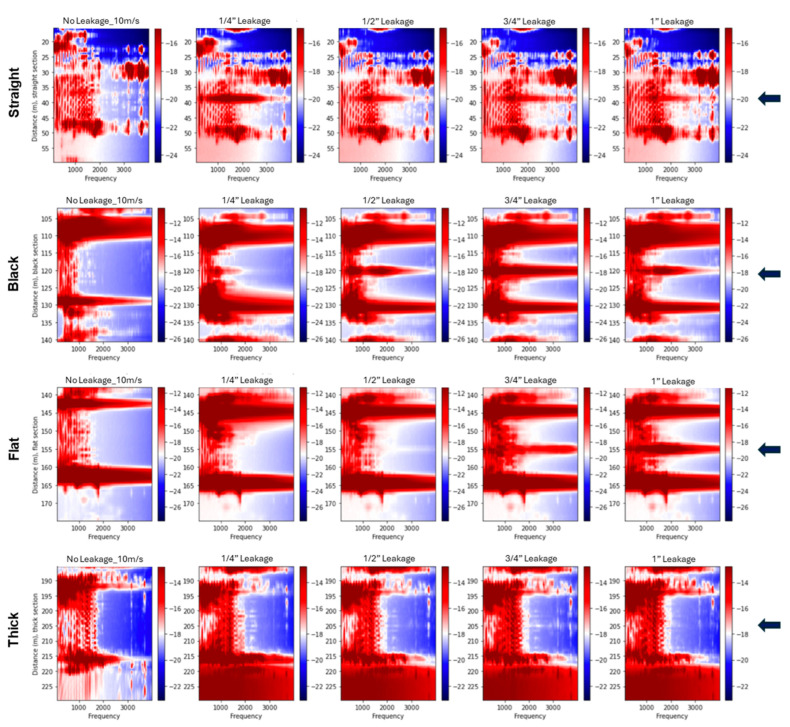
Two-dimensional spectral plots for fiber sections during bottom leakage experiments conducted at a flow velocity of 10 m/s for supported pipeline tests. The rows show data from the straight, black, flat, and thick cables, respectively. Each column corresponds to a different leakage condition, starting with the no-leakage control on the left, followed by increasing hole sizes of ¼”, ½”, ¾”, and 1”. Spectral amplitudes are presented in decibels (dB). The arrows indicate the leakage position.

**Figure 13:**
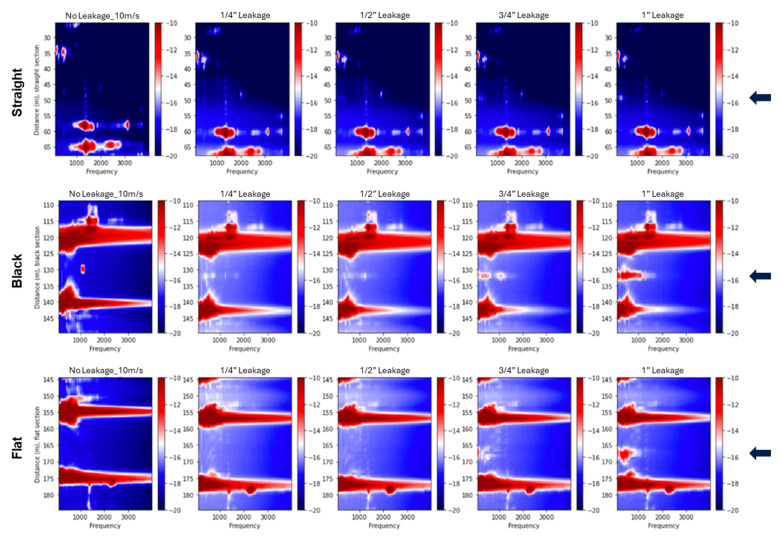
Two-dimensional spectral representations of bottom-positioned leaks recorded at a flow velocity of 10 m/s for buried pipeline tests. The top row presents data from the straight fiber section, the middle row shows results from the black fiber section, and the bottom row displays data from the flat fiber section. From left to right, each column corresponds to the control case (no leakage), followed by progressively larger leak sizes of ¼”, ½”, ¾”, and 1”. Spectral amplitude is expressed in decibels (dB). The arrows indicate the leakage position.

Figure 12 presents the 2D spectra for bottom-positioned leakage cases in the supported pipeline at a fixed flow velocity of 10 m/s. Across the straight, black, and flat cables, high-amplitude spectral anomalies are observed near the leakage points, as indicated by blue arrows. In contrast, the thick cable shows absent responses. Larger leak sizes produce more prominent spectral features, particularly within the 1000–2000 Hz range, where broadband energy with identifiable peak frequencies is observed. The observed spectral patterns vary with cable type, leak size, and orientation. Figure 13 shows the corresponding spectral responses in the buried configuration. In this case, leakage-related anomalies are less distinct, and the spectral energy shifts toward lower frequencies. This suggests a narrower frequency band, likely due to the damping effect introduced by sand confinement. Although the flat cable shows relatively clearer leakage signatures, overall spectral content appears attenuated compared to the supported setup. These observations are consistent with the trend of reduced leakage sensitivity previously noted in the vibration intensity profiles.

**Figure 14:**
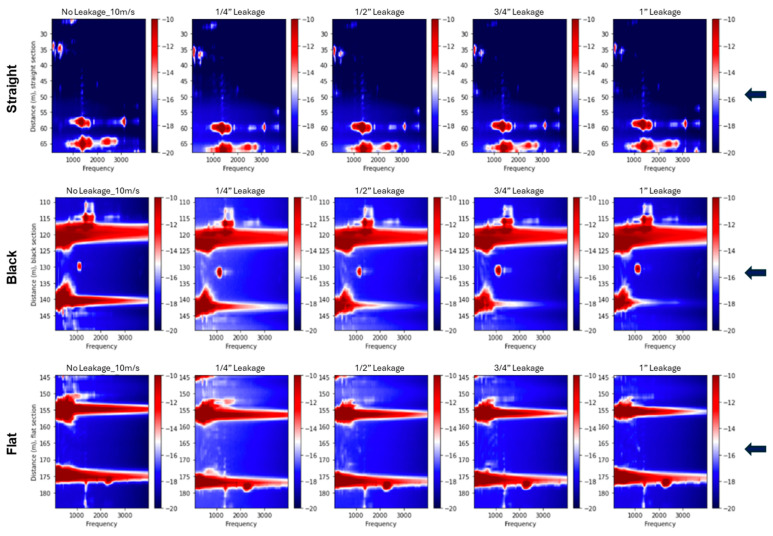
Two-dimensional spectral representations of top-positioned leaks recorded at a flow velocity of 10 m/s for buried pipeline tests. The top row presents data from the straight fiber section, the middle row shows results from the black fiber section, and the bottom row displays data from the flat fiber section. From left to right, each column corresponds to the control case (no leakage), followed by progressively larger leak sizes of ¼”, ½”, ¾”, and 1”. Spectral amplitude is expressed in decibels (dB). The arrows indicate the leakage position.

**Figure 15:**
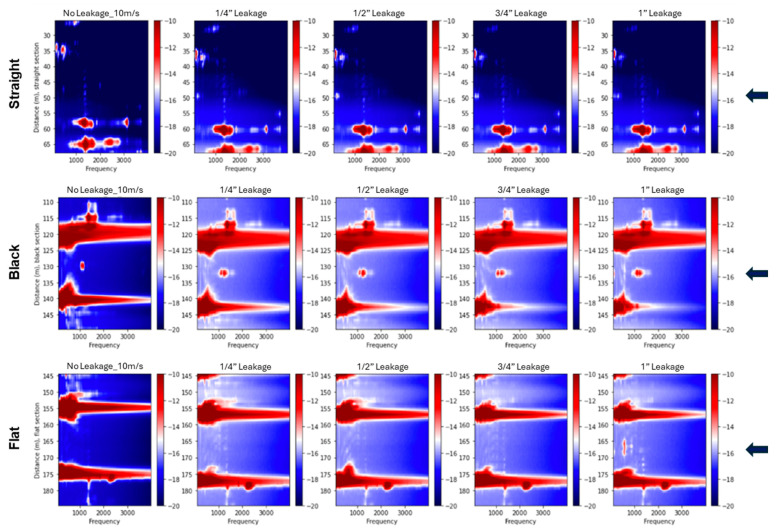
Two-dimensional spectral representations of side-positioned leaks recorded at a flow velocity of 10 m/s for buried pipeline tests. The top row presents data from the straight fiber section, the middle row shows results from the black fiber section, and the bottom row displays data from the flat fiber section. From left to right, each column corresponds to the control case (no leakage), followed by progressively larger leak sizes of ¼”, ½”, ¾”, and 1”. Spectral amplitude is expressed in decibels (dB). The arrows indicate the leakage position.

An interesting phenomenon was observed for the black cable: a prominent peak appeared in a narrow frequency band around 1000 Hz near the leakage point during the control test. This peak was also present in all subsequent leakage tests, as shown in Figure 13 (bottom-positioned leak), Figure 14 (top-positioned leak), and Figure 15 (side-positioned leak). It is suspected that this narrow-band peak is related to a coupling issue near the leakage point, as it persists regardless of leakage conditions. On the other side, leakage events are expected to excite a much broader frequency band, rather than being concentrated at a single narrow frequency, as confirmed in other tests. This further supports the idea that the ~1000 Hz peak might be due to a coupling artifact. If we ignore this narrow frequency peak, the results reinforce the observation from the flat cable: internal cables are most sensitive to bottom leakages, where the fiber cable is closest to the leak source. Referring back to the standard deviation plot (Figure 7) and vibration intensity plots (Figure 9), the relationship between the black cable sensitivity and leakage position is unclear. This is because the standard deviation or the vibration intensity reflects all vibration signals across the frequency spectrum without distinguishing their sources. The high amplitudes observed in the standard deviation and intensity plots for the top and side leakage tests are most likely attributable to coupling issues rather than actual leakage. Therefore, for reliable leakage identification, frequency spectral analysis is also necessary.

In addition, the 2D spectra reveal distinct standing wave patterns and resonant modes propagating along the pipeline. These appear as horizontal spectral bands and are especially prominent in the supported configuration, where the boundary conditions favor the formation of clear vibrational modes. Their visibility across all sensing cables provides valuable insight into the dynamic response of the pipeline structure under flow-induced excitation.

## 4. Discussion

Ensuring pipeline integrity is critical for the safe and efficient transportation of energy and for minimizing environmental risks. Compared to traditional integrity assessment methods such as hydrostatic testing and inline inspection tools, DAS offers distinct advantages, including continuous, real-time monitoring over long distances with minimal operational disruption. Owing to these benefits, research interest in the use of DAS for pipeline leak detection has grown significantly in recent years. However, most existing studies focus on externally deployed fiber-optic cables, which are often costly or impractical for existing pipeline infrastructure.

In this study, we present—for the first time—an investigation into the leak detection capabilities of internally deployed fiber-optic cables. Key questions remain regarding how detectability is influenced by factors such as leak size, leak position, background flow velocity, cable design, and pipeline installation conditions. Through a comprehensive set of controlled experiments, we systematically address these variables and provide new insights into the performance and limitations of DAS for leak detection. Additionally, we implemented several advanced signal processing algorithms to enhance anomaly detection capabilities. The detection performance was evaluated using both time-domain vibration intensity profiles and frequency-domain spectral analysis, providing a comprehensive assessment of the system’s sensitivity and reliability.

Leak size and position were found to play a critical role in detection sensitivity. In the supported pipeline configuration, bottom-positioned leaks produced the clearest vibration anomalies, particularly when the leak size exceeded ½”, primarily due to their proximity to the internally deployed fiber-optic cables. The internal black and flat cables exhibited effective sensitivity at the leak location. This might be attributed to their soft, flexible structure, which allows better coupling with the pipe wall and stronger interaction with leak-induced vibrations. In contrast, the thick internal cable remained largely unresponsive due to its heavy, wireline-like structure, which limited its interaction with leak-induced vibrations. Leak detection at the top and side positions was more challenging across all cables. However, the straight external cable was able to detect top-positioned leaks when the size reached ¾” or larger. This response is attributed to its direct alignment with the leak location and stable mechanical coupling, as it was taped along the top of the pipe.

The overall detectability was reduced when the pipeline configuration changed from supported to buried. The flat cable showed a response only for bottom-positioned leaks with a 1-inch diameter, while the straight cable did not exhibit measurable sensitivity for any tested size or position. These results confirm that burial conditions, particularly soil damping, attenuate the propagation of leak-induced vibrations, limiting the effectiveness of detection.

Flow velocity was found to significantly influence leakage detection sensitivity across both pipeline configurations. In the supported setup, increasing flow velocity enhanced the detectability of leakage-induced vibration anomalies for the straight and flat cables, while the thick cable remained unresponsive. A similar trend was observed in the buried configuration, where the flat cable began detecting anomalies at 10 m/s, and the straight cable responded only at the highest tested flow velocity of 18 m/s. These results confirm that higher gas flow can improve detectability, particularly for less sensitive cables. However, the relationship is not strictly linear. At low flow velocities, the signal produced by the leak may be too weak to rise above the background noise. As flow velocity increases, the signal-to-noise ratio improves, allowing the leak-induced response to become visible. At higher velocities, especially in internal cables exposed to the fluid flow, noise levels can increase more rapidly than the signal itself. This reduces the signal-to-noise ratio and may limit detectability. These observations suggest the existence of an optimal flow range for effective leakage detection. This optimal flow range may depend on the specific pipeline operating conditions and warrants further detailed investigation. Additionally, the relatively short length of the test pipe (21 m) amplifies boundary reflections, increasing background noise near the ends. In real field pipelines, which are much longer, background noise is expected to be lower, and signal-to-noise ratios higher, leading to better overall detection performance in practice.

The frequency-domain analysis using 2D Fourier spectra corroborated the time-domain findings. In the supported configuration, high-amplitude spectral anomalies were clearly localized around the leakage points for the straight, black, and flat cables, particularly at larger leak sizes. These anomalies were most prominent in the 1000–2000 Hz range, where broadband energy with identifiable peak frequencies was observed. The spectra also revealed distinct standing wave patterns and resonant modes, seen as horizontal bands extending across the pipeline, which were especially visible in the supported setup due to favorable boundary conditions. In the buried configuration, leakage-related anomalies were less distinct, and the spectral energy shifted toward lower frequencies, resulting in a narrower frequency band. The internal cables showed a detectable response only for bottom-positioned leaks at the largest size, while the straight cable showed no measurable spectral anomaly. These results support the conclusion that burial conditions attenuate leak-induced vibrations and reduce signal strength and detectability.

Overall, the results demonstrate that leak detection using DAS is influenced by multiple interacting factors, including pipeline installation method, flow rate, leak orientation and size, and cable type and deployment method. The supported configuration produced stronger and more consistent detection signals, whereas burial conditions introduced damping effects that reduced sensitivity. These findings highlight the importance of optimizing fiber deployment strategies and accounting for environmental conditions in DAS-based pipeline monitoring applications.

Nevertheless, several important aspects remain to be thoroughly investigated to enable broader field deployment of DAS-based pipeline monitoring. These include the effects of different types of transported fluids (e.g., liquid hydrocarbons, CO_2_, or multiphase mixtures), fluid properties (such as density and viscosity), various soil types, and burial depths, as well as the impact of ground movement and geohazards. In addition, coupling quality, particularly in internal deployments, needs to be better understood under long-term operational conditions and environmental variability. Future studies should aim to systematically explore these factors through extended field trials, long-duration monitoring campaigns, and advanced data processing to better quantify DAS performance and reliability across a range of realistic scenarios.

## 5. Conclusions

This study assessed the performance of Distributed Acoustic Sensing (DAS) for detecting gas pipeline leaks using multiple fiber cable types deployed under both supported and buried configurations. By combining time-domain and spectral analyses, the results demonstrated that leak detectability depends strongly on leak size, position, flow rate, and fiber deployment method and type.

Among the tested cables, the black and flat internal fibers, which have soft and flexible structures, showed higher sensitivity to leak-induced vibrations in the supported setup. The thick cable remained largely unresponsive due to its rigid, wireline-like design. The straight external cable demonstrated improved performance when mechanically coupled and aligned with the leak. Increasing flow velocity generally enhanced detectability, although a reduction in signal-to-noise ratio was observed at higher velocities due to increased background noise. The internal cable exhibits the highest sensitivity to leakage occurring at positions closest to the fiber optic cable. In the buried configuration, detection sensitivity was significantly reduced across all cables due to soil-induced damping effects. Data analysis also indicates that spectral analysis is essential for distinguishing vibration signals from different sources, making it an important tool for reliable leakage detection.

These findings highlight the practical considerations in applying DAS for pipeline monitoring and suggest that optimizing cable selection, mechanical coupling, and accounting for environmental conditions are essential for improving leak detection performance.

## Figures and Tables

**Table 1:** Start and end positions of each fiber segment in the supported and buried pipeline configurations. The thick cable was not included in the buried setup. Flow directions indicate the direction of gas propagation relative to each fiber segment.

Cable Type	Supported Pipe (m)	Buried Pipe (m)	Flow Direction
Straight (taped external)	32 → 52	35 → 58	Downstream
Black (internal)	110 → 131	118 → 140	Upstream
Flat (internal)	143 → 164	154 → 175	Downstream
Thick (internal)	192 → 215	Not included	Upstream

## Data Availability

The data presented in this study may be available on request from the corresponding author.
